# Eczema Produced by Tincture of Arnica

**Published:** 1876-01

**Authors:** 


Eczema Produced by Tincture of Arnica.—Dr. Whittaker has (*Ohio Clinic*) lately encountered several cases illustrating the evils of the local application of tincture of arnica. One young man with orchitis applied it, and came to Dr. Whittaker with an extensive and profound eczema, which lasted for three weeks. Another man, having let a ten-pin ball fall upon his toes, applied it to relieve the pain, and had a violent eczema, which was not cured for two weeks. At the society at which this was read, Dr. Longworth mentioned the fact as something peculiar about this drug; the majority of persons may use it with impunity, but it will now and then act as a virulent poison, producing an eczema not limited to the seat of its application, but which becomes universal, and is often very obstinate to treatment.—*American Med. Weekly.*

